# Appropriate use of antimicrobial therapy for COVID-19 co-infection

**DOI:** 10.2217/imt-2021-0134

**Published:** 2021-06-11

**Authors:** Chih-Cheng Lai, Wen-Liang Yu

**Affiliations:** ^1^Department of Internal Medicine, Kaohsiung Veterans General Hospital, Tainan Branch, Tainan, Taiwan; ^2^Department of Intensive Care Medicine, Chi Mei Medical Center, Tainan, Taiwan; ^3^Department of Medicine, School of Medicine, College of Medicine, Taipei Medical University, Taipei, Taiwan

**Keywords:** antimicrobial agents, *Aspergillus*, bacteria, co-infection, COVID-19, mucormycosis, respiratory virus

Since the first outbreak of SARS-CoV-2 in Wuhan, China, COVID-19 has rapidly spread worldwide and has become a global pandemic [1]. As of 26 May 2021, there have been more than 167 million confirmed cases of SARS-CoV-2 infections, including 3.48 million deaths [2]. The clinical presentations of COVID-19 are protean, including fever, respiratory tract symptoms and extra-respiratory manifestations, including cardiac, gastrointestinal, hepatic, renal, neurological, olfactory, gustatory, ocular, cutaneous and hematological symptoms. Moreover, SARS-CoV-2 infections can manifest as asymptomatic, acute respiratory disease, pneumonia and critically ill diseases [3]. Moreover, the pathogenesis of SARS-CoV-2 in the lung tissue could create favorable environmental conditions for invasion of other micro-organisms to develop co-infectious diseases. In addition to SARS-CoV-2 itself, co-infection with other respiratory viruses, bacteria and fungi have been reported, particularly for the patients with severe COVID-19 diseases. Therefore, how to appropriately use non-anti-SARS-CoV-2 antimicrobial agents in the management of COVID-19 patients should be another serious concern during this pandemic.

## Epidemiology

Many studies reported the prevalence of co-infection among COVID-19 patients; however, the estimated prevalence varied and ranged from 1.4 to 57% [4]. The huge variation could be caused by different study population, type of copathogens and study sites. Recently, Musuuza *et al.* conducted a systematic review and meta-analysis of 118 articles to investigate the occurrence of co-infections and their outcomes among patients with SARS-CoV-2 infections [4]. They found that the pooled prevalence of co-infection was 19% (95% CI: 14–25%) and the prevalence of co-infection was highest for non-ICU patients at 29% (95% CI: 14–46%), followed by 18% (95% CI: 12–25%) among combined ICU and non-ICU patients and 16% (95% CI: 8–25%) among only ICU patients [4]. In contrast, a case–control study revealed that patients with cobacterial infection had greater proportion of severe/critical disease at presentation than those without co-infection (80 vs 30%; p < 0.001) [5]. Most importantly, patients with a co-infection had a prolonged length of hospital stay with an average of 29 ± 6.7 days and carried a higher risk of death than those without co-infection (odds ratio [OR]: 2.84; 95% CI: 1.42–5.66) [4] as previously reported [5,6].

According to the type of copathogens, the prevalence of viral co-infections was highest (10%; 95% CI: 6–14%) followed by bacterial (8%; 95% CI: 5–11%) and fungus (4%; 95% CI: 2–7%) [4]. *Klebsiella pneumoniae* (9.9%) was the most common identified bacteria, followed by *Streptococcus pneumoniae* (8.2%) and *Staphylococcus aureus* (7.7%). Among coviral infection, influenza type A (22.3%) was the most frequently identified viruses followed by influenza type B (3.8%), and respiratory syncytial virus (3.8%). Among cofungal infection, *Aspergillus* (6.7%) was the most common fungi, followed by *Candida* spp. (1.0%) and mucormycosis (0.3%). In addition to the above common organisms, many other organisms have been reported as copathogens, including bacteria, such as *Haemophilus influenzae*, *Escherichia coli*, *Stenotrophomonas maltophilia*, *Bordetella*, *Moraxella catarrhalis*, *Pseudomonas* spp., *Enterococcus faecium*, *Mycoplasma pneumoniae*, *Chlamydia pneumoniae*, *Legionella pneumophila*, *Acinetobacter* spp. and *Mycobacterium tuberculosis*; and viruses such as non-SARS-CoV-2 coronavirus, rhinovirus/enterovirus, adenovirus, parainfluenza, metapneumovirus and HIV [4,7]. Moreover, several antimicrobial resistances have been reported among these copathogens, such as extended-spectrum β-lactamase-producing *K. pneumoniae*, carbapenem-resistant New Delhi metallo-β-lactamase-producing *Enterobacterales*, *Acinetobacter baumannii*, methicillin-resistant *S. aureus*, pan-echinocandin-resistant *Candida glabrata* and multitriazole-resistant *Aspergillus fumigatus* [8].

In summary, the risk of co-infection among COVID-19 patients cannot be ignored and the occurrence of co-infection with multidrug resistant organism is possible. Early diagnosis of co-infection and detection of copathogen should be the key to the prompt and appropriate use of antimicrobial therapy.

## Diagnosis

Because patients with co-infection could share the similar clinical presentation as those without co-infections, no clinical rule could accurately identify co-infections among COVID-19 patients. Although procalcitonin (PCT) has been considered a useful biomarker in diagnosing bacterial infections, a retrospective analysis of 60 critical COVID-19 cases did not found the significant difference between peak PCT levels of patients with positive and negative bacterial cultures [9]. Another case–control study showed the same thing – no difference was observed between PCT levels in COVID-19 patients with and without cobacterial infections (p = 0.883) [5]. Moreover, elevation of PCT level may be measured in COVID-19 patients without cobacterial infections and may result in receipt of unnecessary antibiotics. Therefore, the usefulness of PCT in identifying cobacterial infection is limited and further study is warranted to develop accurate prediction model or diagnostic method for co-infection among COVID-19 patients. At present, aggressive investigation of microbial patterns of infectious complications in critically ill COVID-19 patients is what we can do and is also required. In addition to conventional and time-consuming culture methods, several syndromic multiplex PCR panels, such as QIAstat-Dx^®^ Respiratory 2019-nCoV Panel, (Qiagen, The Netherlands) and Biofire FilmArray RP-2.1 (Biofire FilmArray Respiratory Panel-2 plus SARS-CoV-2; bioMeriaux, France) had incorporated SARS-CoV-2, to help us simultaneously identify SARS-CoV-2 and other commonly encountered respiratory pathogens. Under the help of these SARS-CoV-2 containing syndromic/co-infection tests, clinicians can promptly detect the possible copathogens and the use of rapid multiplex PCR assay can exhibit positive impact on antibiotic stewardship program by administering appropriate antibiotics earlier and avoiding unnecessary prescriptions [10].

## Antibiotic overuse

Although a significant portion of COVID-19 patients had co-infection requiring additional antimicrobial agents, much more patients without co-infection had also been prescribed antibiotics [11]. In the early 2020, Chen *et al.* reported that more than 70 and 15% of 99 patients with COVID-19 in Wuhan, China had received antibiotic and antifungal agents, respectively [12]. Another study of 138 hospitalized COVID-19 patients reported that moxifloxacin – the most used antibiotic, was prescribed in 89 (64.4%) patients [13]. Further large-scale study of 1099 patients in China still reported that 58% received intravenous antibiotics [14]. Even in Brazil, Teich *et al.* reported that 84.7% of 72 hospitalized COVID-19 patients had received intravenous antibiotic therapy [15]. Overall, a meta-analysis of 154 studies by Langford *et al.* presented that 74.6% (95% CI: 68.3–80.0%) of COVID-19 patients had received antibiotics but the estimated bacterial co-infection was only 8.6% (95% CI: 4.7–15.2%) [16]. Moreover, the prevalence of antibiotic prescribing increased with the patient age (OR: 1.45; 95% CI: 1.18–1.77) and was higher among patients requiring mechanical ventilation (OR: 1.33; 95% CI: 1.15–1.54) [16]. In fact, one study even showed that 64% of COVID-19 patients without co-infection were administered antibiotics despite the absence of bacterial coinfection or secondary infection [5]. All the above findings indicated that the inappropriate use of antibiotic during COVID-19 pandemic is obvious [17]. Because antibiotic overuse can result in the increasing consumption of antibiotics and cause the collateral damage – increasing antimicrobial resistance [18,19], how to avoid the inappropriate use of antimicrobial agents has become another great challenge. Continuing implementation of antibiotic stewardship program in optimizing the antimicrobial therapy among hospitalized COVID-19 patients could be a possible solution. In the meanwhile, many other components such as diagnostic stewardship, early risk assessment and infection prevention and control of strategies of COVID-19 mitigation should be integrated into this comprehensive program [20].

## Recommendation

In addition to target antimicrobial therapy after identification of copathogens, how to optimize the empirical use of non-anti-SARS-CoV-2 antimicrobial therapy should be the most difficult component of antibiotic stewardship program. Herein, we reviewed several treatment guidelines but not all guidelines had issued the use of antibiotic in the COVID-19 patients. The Surviving Sepsis Campaign on the management of critically ill adults with COVID-19 suggests using empiric antimicrobials/antibacterial agents better than no antimicrobials in mechanically ventilated patients with COVID-19 and respiratory failure [21]. However, the antimicrobial therapy should be assessed for de-escalation and the duration of therapy and spectrum of coverage should be re-evaluated based on the microbiology results and the patient’s clinical status [21]. According to the recommendation of the National Institutes of Health, US, they recommended against the use of antibacterial therapy (e.g., azithromycin, doxycycline) in the absence of another indication for managing outpatients with COVID-19 (AIII) [22]. In contrast, there is insufficient data to recommend empiric broad-spectrum antimicrobial therapy in the absence of another indication for the patients with severe or critical COVID-19 but once initiated, their use should be reassessed daily in order to minimize the adverse consequences of unnecessary antimicrobial therapy [22]. The Dutch Working Party on Antibiotic Policy provided an evidence-based recommendation for the use of antibacterial therapy in adults with COVID-19 having the following suggestions: restrictive use of antibiotics in patients with proven or a high likelihood of COVID-19, especially for mild to moderately ill patients upon admission; antibiotic therapy should be considered if the clinician has a high suspicion of bacterial co-infection in a patient with compatible radiological findings and/or inflammatory biomarkers and those who are severely immunocompromised; the selection of the empirical antibiotics in case of suspected bacterial co-infection could be based on the disease severity and local/national guidelines [23]. In summary, most of the guidelines or recommendations regarding this issue were based on low-quality evidence. Before further study can provide high-quality evidence-based recommendation, the use of empirical antimicrobial therapy could be considered in severe or critical COVID-19 patients but clinicians should conduct daily assessment for de-escalation thereafter.

## Conclusion

How to appropriately use non-anti-SARS-CoV-2 antimicrobial therapy for COVID-19 patients with co-infection remains a complicated issue due to: the variable prevalence and microbiological distribution of co-infections; difficult to differentiate patients with or without co-infections; and lack of strong evidence. However, the occurrence of co-infection is possible for COVID-19 patients and can be associated with a high morbidity and mortality, especially for severe or critical ill patients. Appropriate use of antimicrobial agents according to antibiotic stewardship program could be the best practice right now.

## Future perspective

COVID-19 is a new global pandemic and co-infection with bacteria, virus and fungus is possible. However, many aspects regarding COVID-19 co-infection remain unclear. Further study is warranted to investigate how many COVID-19 patients would get co-infection, what are the copathogens and what are the accurate biomarkers to diagnose co-infection. An antibiotic stewardship program would play a critical role in the management of COVID-19 patients with co-infections.
